# Histone 3 lysine 9 acetylation is a biomarker of the effects of culture on zygotes

**DOI:** 10.1530/REP-17-0112

**Published:** 2017-07-04

**Authors:** C Rollo, Y Li, X L Jin, C O’Neill

**Affiliations:** Human Reproduction UnitKolling Institute Sydney Medical, School University of Sydney, Sydney, Australia

## Abstract

Acetylation of histone proteins is a major determinant of chromatin structure and function. Fertilisation triggers a round of chromatin remodelling that prepares the genome for the first round of transcription from the new embryonic genome. In this study we confirm that fertilisation leads to a marked progressive increase in the level of histone 3 lysine 9 acetylation in both the paternally and maternally derived genomes. The culture of zygotes in simple defined media caused a marked increase in the global level of acetylation and this affected the male pronucleus more than the female. The culture created a marked asymmetry in staining between the two pronuclei that was not readily detected in zygotes collected directly from the reproductive tract and was ameliorated to some extent by optimized culture media. The increased acetylation caused by culture resulted in increased transcription of *Hspa1b*, a marker of embryonic genome activation. Pharmacological analyses showed the hyperacetylation of H3K9 and the increased expression of *Hspa1b* caused by culture were due to the altered net activity of a range of histone acetylases and deacetylases. The marked hyperacetylation of histone 3 lysine 9 caused by culture of zygotes may serve as an early biomarker for the effects of culture on the normal function of the embryo. The results also provide further evidence for an effect of the stresses associated with assisted reproductive technologies on the normal patterns of epigenetic reprogramming in the early embryo.

## Introduction

Activation of gene expression from the new embryonic genome created at fertilisation is an essential requirement for normal development. This is a progressive process that, in the mouse, commences towards the end of the first cell cycle, and reaches maturity by the 8-cell stage (Latham & Schultz 2001). The new transcriptome created by this process has the essential roles of supporting the normal cellular functions present in all cells but also has the unique feature of converting the transcribed genome of the terminally differentiated gametes into the totipotent state of the early embryo. It is generally considered that conversion to totipotency requires a process of extensive epigenetic reprogramming of the new embryonic genomes inherited from the gametes, although there is currently a rather limited understanding of the essential details of this process.

The remarkable capacity of the oocyte’s cytoplasm to reprogramme somatic nuclei to totipotency (following experimental cloning procedures) (Campbell *et al*. 1996) indicates that the oocyte has unique properties required for the generation of this state. There is evidence that control of nuclear histone acetylation is an important component of this reprogramming machinery. For instance, the efficiency of reprogramming after cloning is commonly quiet low, but treatment with a histone deacetylase inhibitor markedly improves its efficiency. Furthermore, this effect persists into subsequent generations of reprogramming (Wakayama *et al*. 2013).

Histone proteins package DNA into nucleosomes, which are the primary unit of organisation of the genome. Nucleosomes consist of two copies each of histones 2A, 2B, 3 and 4. There are a large number of documented post-translational modifications to these proteins which have the effect of further modifying the organisation of chromatin (Kouzarides 2007, Lee *et al*. 2010). Acetylation of histones can profoundly alter chromatin conformation and gene expression. It typically results in chromatin having a more open conformation and this allows the transcriptional machinery and regulatory components to more readily access target DNA sequences (Grunstein 1997, DesJarlais & Tummino 2016). Steady-state histone acetylation levels are maintained by the coordinated actions of a complex array of histone acetylases (HATs) and deacetylases (HDACs), so acute changes in acetylation levels can result from changes in the balance between HAT and HDAC activities (Grunstein 1997).

The role of histone modifications in embryonic genome activation has received some experimental attention. RNAs encoding a range of HDACs are present within the early embryo (Ma & Schultz 2008). Functional analysis shows that HDAC1 activity is sufficient for the synthesis of RNA polymerase II-dependent transcripts in the early embryo (Ma & Schultz 2008). HDAC1 targets the acetylation of a range of targets, including the lysine at position 9 of histone 3 (H3K9), H3K14, H4K5 and H3K8 (Dovey *et al*. 2010). Acetylation of H3K9 and H3K14 is commonly enriched at epigenetically bivalent promoters (Karmodiya *et al*. 2012), i.e. promoters that have an accumulation of both activating and repressive histone modifications. Bivalency of promoters of many genes critical to lineage specification is a characteristic of pluripotent cells (Wong *et al*. 2014).

Acetylation at H3K9 (H3K9ac) is not detected in oocytes at meiosis II but becomes abundant in all stages of preimplantation development (Wang *et al*. 2007, Bošković *et al*. 2012). After fertilisation it becomes equally abundant in both the male and female pronuclei of the mouse zygote (Bošković *et al*. 2012). Fertilisation by intracytoplasmic sperm injection shows that H3K9 acetylation occurs approximately 6 h after sperm injection (Wang *et al*. 2007). This chronology is recapitulated after somatic nuclei are transplanted to oocytes, where they become de-acetylated within 1 h of transfer and re-acetylated 6–10 h after oocyte activation (by which time nuclei become decondensed) (Wang *et al*. 2007). These findings indicated that, in the oocyte, the steady state favours a net de-acetylating activity which tips to a net acetylating state some hours after fertilisation (or oocyte activation).

Transcription, as assessed by BrUTP incorporation into RNA, can be readily detected in zygotes (Aoki *et al*. 1997) and occurs at levels approximately 40% of that observed in the 2-cell embryo. Plasmid studies show that transcription in the zygote is not dependent upon enhancers, but in the 2-cell embryo enhancers become important for optimal transcription (Wiekowski *et al*. 1991, Majumder *et al*. 1993). It is considered that promoters become strongly repressed by cytoplasmic factors in 2-cell embryos and the activity of enhancers is required to lift this repression (Henery *et al*. 1995). Histone acetylation seems to play a role in this change since increasing histone acetylation increased enhancerless promoter expression 20-fold, but only increased enhancer-driven expression 3-fold in 2-cell embryos (Wiekowski *et al*. 1993). Furthermore, the expression of canonical transcriptional markers of genome activation (Davis *et al*. 1996) and the incorporation of BrdUTP into RNA (Aoki *et al*. 1997) are increased in 2-cell stage embryos when histone acetylation is increased by treatment with a HDAC inhibitor (Tokoro *et al*. 2010). These lines of evidence implicate histone acetylation as an important component of activation of transcription from the embryonic genome.

The expression of *Hspa1b* (also known as *Hsp70.1*) is accepted as one landmark of the initial activation of gene expression in the zygote (Christians *et al*. 1995) and can serve as a convenient tool for interrogating the processes of embryonic genome activation. *Hspa1b* transcription begins during G2-phase of the zygotic cell cycle and further increases in the G1-phase of the 2-cell embryo. This first wave of *Hspa1b* transcription is independent of new protein synthesis inferring that the basic requirements for transcription pre-exist within the zygote. Interestingly, *Hspa1b* mRNA in 2-cell embryos collected directly from the reproductive tract was 15 times less than that from the equivalent 2-cell embryos cultured *in vitro* from the 1-cell stage (Christians *et al*. 1995). Furthermore, *Hspa1b*-promoter-driven luciferase activity was 5-fold higher in 2-cell embryos cultured *in vitro* from the 1-cell stage, compared to the equivalent 2-cell embryos collected directly from the reproductive tract of females (Christians *et al*. 1995). This impact of embryo culture on the expression of *Hspa1b* provides a further, clinically relevant, tool that can be exploited for understanding the control of embryonic genome activation.

There is a growing body of evidence implicating alterations to the early embryo’s growth environment in perturbing the normal processes of epigenetic reprogramming, which in turn may result in maladaptive lifelong changes to the organism’s homeostasis. Culture of the preimplantation embryo is one such perturbation. Culture from the zygote to blastocyst stage followed by embryo transfer caused changes in the postnatal growth rates and allometry of some organs. Importantly this effect persisted into the next generation indicative of an epigenetic basis (Mahsoudi *et al*. 2007). A powerful proof of the epigenetic basis of the effects of embryo culture conditions was evidence that zygote culture caused incomplete reprogramming of metastable epialleles. Both the canonical *Agouti viable yellow (Avy)* and *Axin1^Fu^* metastable epialleles show variable expressivity in progeny and this has an epigenetic basis (Rakyan *et al*. 2002). In their expressed state both alleles cause abnormalities in progeny and this is associated with reduced DNA methylation of the epialleles (Rakyan *et al*. 2002, Morgan *et al*. 2008, Fernandez-Gonzalez *et al*. 2010). Zygote culture increased the expression of these epialleles, resulting in an increase in abnormalities of progeny (Morgan *et al*. 2008, Fernandez-Gonzalez *et al*. 2010). The increased expressivity of the epialleles in progeny after culture was associated with change levels of H3K9ac and H3K4 dimethylation associated with the allele, while inhibition of HDACs in the zygote increased the extent of the epigenetic lability of *Axin1^Fu^* (Fernandez-Gonzalez *et al*. 2010). Such observations indicate that some aspects of zygote culture can disturb the processes of epigenetic reprogramming in the early embryo and that remodelling of histone modifications, including H3K9ac, is an important component of this reprogramming process.

It is of interest therefore to understand the effects of culture on the processes of epigenetic modifications to the early embryo and their impacts on the normal onset of gene expression. In this study we confirm the progressive global increase in H3K9ac levels of both the paternally and maternally inherited genomes after fertilisation, with a marked increase from mid-cell cycle. Culture of zygotes *in vitro* led to higher total levels of H3K9ac across the pronuclei and this was more apparent within male pronuclei. These changes depended upon the actions of histone acetylases and histone deacetylases, and resulted in increased transcription from the embryonic genome. The results show that the onset of transcription of a marker of embryonic genome activation is in part regulated by rising level of global histone acetylation, and this process is perturbed by the culture of embryos *in vitro*.

## Materials and methods

Animals were housed and bred in the Kearn’s Animal House (Royal North Shore Hospital, St Leonards). Animal use was according to the Australian Code of Practice for the Care and Use of Animals for scientific purposes and was approved by the Institutional Animal Care and Ethics Committee. Hybrid embryos were collected by mating F1 mice (C57BL/6J X CBA/He). Female mice were housed in groups of 5 and the male mice were housed singularly under a 12 h (h) light/darkness cycle and had food and water *ad libitum*.

Female mice were superovulated with 5 IU equine chorionic gonadotrophin (eCG) (Lyppard chemicals; Sydney, NSW, AUS) and human chorionic gonadotrophin (hCG) (Lyppard) and mated with a male stud of the same strain overnight. Embryos were collected in 37°C hydroxyethylpiperazine-N′-2-ethanesulfonic acid (HEPES) buffered modified human tubal medium (O’Neill 1997), containing 3 mg bovine serum albumin (BSA)/mL (Sigma-Aldrich Company). Embryos were cultured in glutamine and EDTA-modified human tubal fluid media (GE-HTFM) with BSA (3 mg/mL) (O’Neill 1997). In some experiments, KSOM plus amino acid media was used (Biggers & Racowsky 2002).

One-cell embryos were recovered from the oviduct at the times indicated in each experimental design and treated with hyaluronidase from bovine testes – type VIII (Sigma-Aldrich, H-3757) at 37°C for 2–3 min (min) to remove any remaining cumulus cells. They were then either cultured *in vitro* with various treatments or processed immediately for immunofluorescence.

Embryos were cultured in groups of 10 in 10 μL media in 5% CO_2_ in air and 37°C for 2–8 h. All cultured embryos were collected from the reproductive tract at 16.5 h post hCG injection (just after pronuclei formation). For all culture treatment involving pharmacological enhancement, embryos cultured in the absence of chemical inhibitors served as controls.

Embryos were classified in pronuclear stages 1–5 (PN1–5) according to their size and location in the cytoplasm using criteria previously reported (Adenot *et al*. 1997). PN1 zygotes were denoted as the least mature pronuclei that are small and located at the periphery of the oocyte. The most mature pronuclei were classified at PN5 and were large and closely apposed with each other. The paternal pronucleus was identified as the larger and the maternal pronucleus as the smaller and located closest to the polar bodies (Bouniol *et al*. 1995). As mating is presumed to occur 12–13 h post hCG injection (Bouniol *et al*. 1995) embryos were collected at 16.5, 18.5, 21.5 (F1) or 24.5 (B6) h post hCG injection. At these times most zygotes were at stages PN1/2, PN3–4 and PN4–5, respectively.

### Treatments

Broad-spectrum inhibition of histone deacetylase (HDAC) used Trichostatin A (TSA) prepared as 300 µM stock solution in DMSO (Sigma, D2650) and diluted for use in GE-HTFM. The following histone acetylase (HAT) inhibitors were used: KG501 (Fluka, 70485) prepared in GE-HTFM; anacardic acid (AA) (Sapphire Bioscience Pty Ltd, Waterloo, NSW, Australia); Garcinol (Sapphire Bioscience); Butyrolactone 3 (Butyrolactone, Sapphire Bioscience) (all prepared as stock solutions in DMSO and stored at −20°C prior to dilution to working concentration in GE-HTFM on day of use); and Nu 9056 (Tocris Bioscience, Bristol, BS11 0QL, U.K.) prepared directly into GE-HTFM.

### Gene expression analysis

RT-PCR and real-time quantitative RT-PCR (qRT-PCR) were performed as described previously (Jin & O’Neill 2014). Mouse brain RNA was extracted using RNeasy Mini kit (Qiagen, Cat. No.74104). The fresh and cultured zygotes were washed in cold PBS 3 times and transferred individually in minimal volume into RNA lysis buffer. RNA was extracted by freezing three times in liquid nitrogen and thawing with vortexing. DNA was degraded by treatment with the RQ1 RNase-free DNase kit (Promega).

Reverse transcription was performed with 2.5 µM random decamers (Life Technologies). Amplification of cDNA was performed with TaqMan gene expression assays. Target genes and their assay IDs were *Hspa1b* (Mm03038954_s1) and *Actb* (Mm01205647_g1) (Life Technologies). cDNA equivalent to half zygote for Hspa1b and another half for *Actb* was amplified by PCR: 10 min at 95°C, 40 cycles of 15 s at 95°C and 1 min at 60°C on Stratagene MXPro-Mx3000P (Agilent Technologies). Positive controls were the expression of target genes in the mouse brain (cDNA equivalent to 20 ng brain). Negative controls for all reactions were the absence of reverse transcriptase or the RNA sample. Ct values were calculated by the system software. The Delta Ct was a measure of relative changes in the transcripts of the tested gene content of the embryo to housekeeper gene *Actb*. A plot of the relatively normalised expression (2^−(normalised Delta Ct)^) was shown.

### Immunofluorescence

Embryos were washed in PBS (Sigma) for 10 min at 20°C and fixed in 4% (w/v) paraformaldehyde for 30 min at 20°C. They were permeabilized in 0.5% (w/v) Tween 20 and 0.5% Triton X-100 (both from Sigma) solution for a further 30 min at 20°C and washed in PBT (1× PBS + 0.03% Tween 20) for 15 min at 20°C. Blocking of non-specific staining used 30% (v/v) goat serum for 2 h at 20°C followed by staining with primary antibody, anti-histone 3 acetylation K9 rabbit polyclonal antibody (Abcam, ab4441; Sapphire Bioscience Pty Ltd, Sydney, NSW, Australia) (Egelhofer *et al*. 2011) or non-immune rabbit polyclonal IgG (control) (Sigma) 18 h at 4°C. Optimal antibody concentration was determined by comparing antibody dilutions of 1:100, 1:200, 1:400 and 1:800. A dilution of 1:100 had the optimal signal-to-noise ratio when compared with non-immune control used throughout the study. Embryos were then washed in 1xPBT at 20°C and incubated in the secondary antibody/1× PBT (1:250), (Anti-rabbit IgG, F(ab)_2_ fragment FITC raised in goat, Sigma, F1262) in darkness for 1 h at 20°C. Following washing with 1× PBT they were counterstained with either 0.1 µg/mL propidium iodide (PI added to Vectashield H-1000 Sigma, 56H3612) or DAPI 1.5 µg/mL (in Vectashield H-1200).

Imaging used epifluorescence on a Nikon Optiphot microscope (Nikon, Japan) or confocal microscopy (Leica TCS SP5, Leica Microsystems Pty Ltd, Australia). Images from conventional microscopy were captured using NIS-Elements F 3.2 (Version F 3.2, Nikon Instruments Inc.; Melville, NY, USA). Confocal images used a Leica Plan Apo 63X/1.4 oil objective and had an optical section thickness of 1.13 µm and were processed using LAS AF software (1.3.1 build 525, Leica Microsystems). Images were shown as either single equatorial optical sections of each pronuclei or Z-stacks of sequential optical sections throughout the full depth of each pronuclei.

Nucleolus precursor bodies (NPBs) were manually counted from conventional microscope’s single equatorial images. Quantification of the intensity of fluorescence used Image Pro Plus software (version 6.3, Media Cybernetics Inc.). Each pronucleus was measured individually for cross-sectional area (at its equatorial section). The total and mean optical density of H3K9ac staining across the equatorial section of each pronuclei was measured and recorded as the optical density of fluorescence staining per pixel. Where longitudinal temporal comparisons of staining or the effect of drug treatment were analysed, embryos from each time point or treatment group were processed at the same time with identical microscope and image capture and processing conditions.

SPSS statistical package (version 20, SPSS) was used for all statistical analyses. Univariate analysis of all continuous variables was used to determine the significance of the main factors (treatment, pronuclear stage, and parental origin of the pronuclei) and for any interaction effect between the main factors. The least significant difference (LSD) *post hoc* multiple comparisons test was used in some cases to identify significance between individual groups. Experimental replicate number was included into the statistical model as a covariant and was included into the final analysis if it resulted in an improved model.

## Results

**Figure 1:**
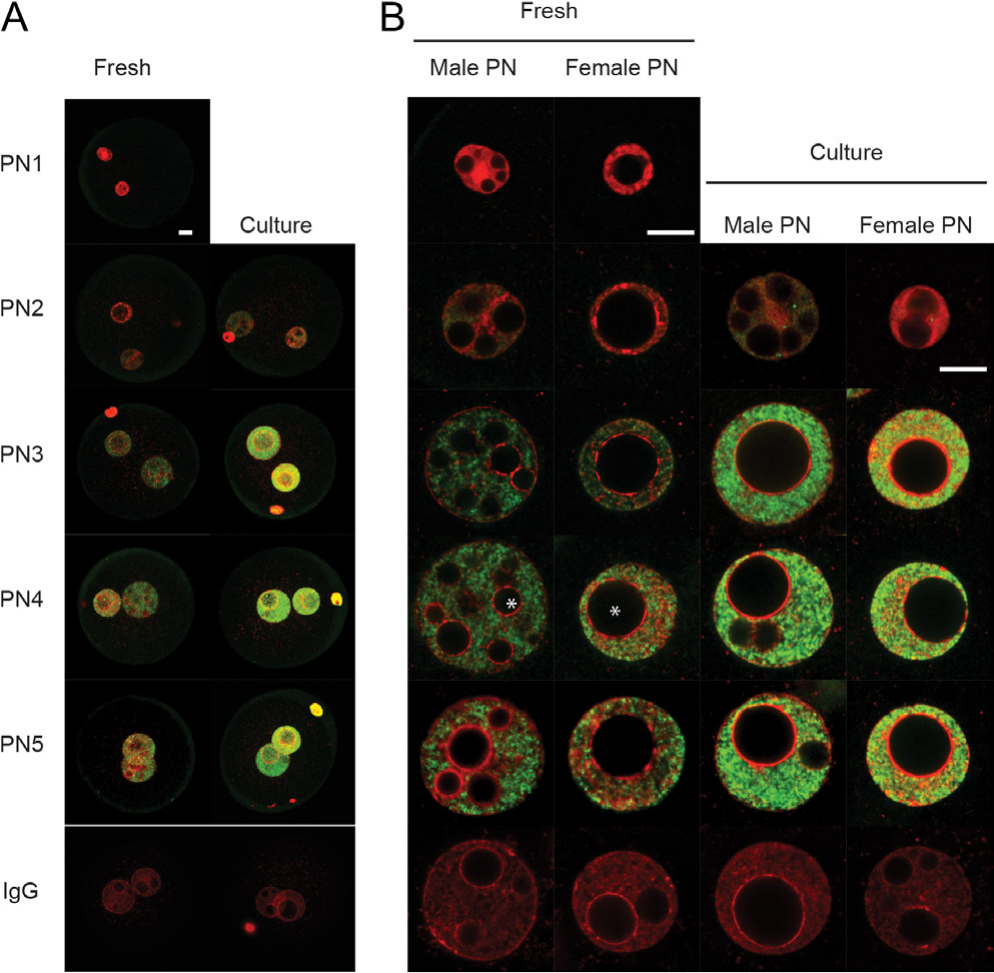
Global H3K9 acetylation levels in the male and female pre-nuclei of zygotic maturation *in vivo* (Fresh) and during culture *in vitro*. (A) Representative images of z-stack confocal projections of merged images for staining of H3K9ac (green) and DNA (PI, red). Staining was performed on each stage of zygote development PN1–5. Non-immune IgG served as a control. (B) Single confocal sections (1.13 µm) through the equatorial plane of the male and female PN of fresh zygotes, and cultured zygotes. White * show examples of nucleolus precursor bodies (NPBs) within the pronuclei. The images are representative of 5 replicates with at least 5 embryos per replicate. Scale bar represents 10 µm.

Confocal projections (Fig. 1A) of zygotes collected directly from the oviduct showed that H3K9ac staining was not detected in PN1 stage zygotes and was at a very low level at the PN2 stage in both pronuclei. Staining increased in PN3 stage zygotes and further increased at subsequent stages of zygotic maturation in both pronuclei. The differences in size and structural complexity of the male and female pronuclei make direct comparisons in staining levels challenging, but consistent differences in staining intensity between the two pronuclei were not obvious in fresh zygotes (Fig. 1A). Larger individual equatorial confocal sections (Fig. 1B) showed staining was distributed throughout much of the nucleoplasm at each stage of maturation. The staining pattern was more heterogeneous within the nucleoplasm of the female pronucleus. Staining was excluded from the NPBs and was low in the pericentric heterochromatin ring that surrounds each NPB, and was also lower at the periphery of the pronuclei.

**Figure 2:**
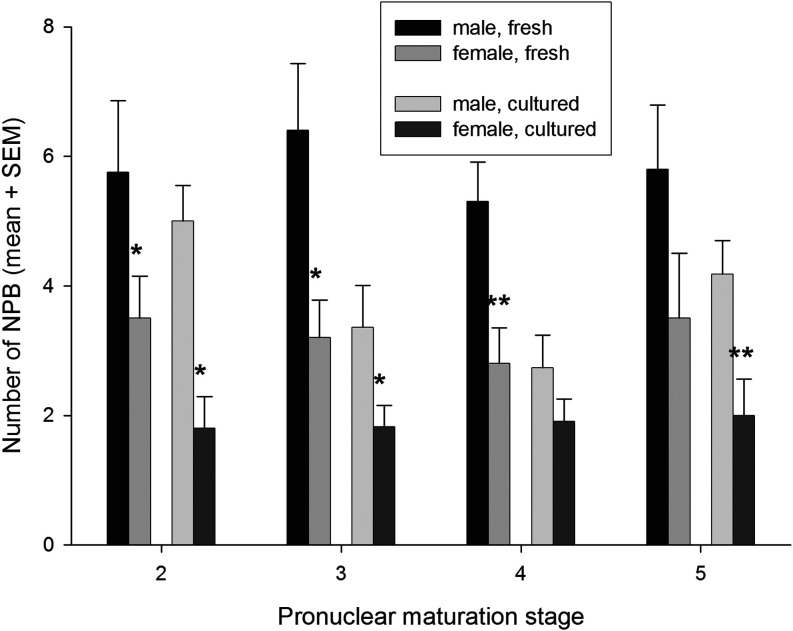
The effect of culture *in vitro* on the number of NPB observed in each pronuclei. Analysis of variance showed that there was no overall effect of zygote maturation stage on the number of NPBs for either the male or female zygote (*P* > 0.05) and there was a significant overall effect of culture (*P* < 0.0001) and parent-of-origin of the NPB (*P* < 0.0001). There were, however, no statistically significant interaction effects between maturation stage, gender and culture on the NBP. This shows that the effects of culture and parent-of-origin of the pronuclei were independent effectors of the number of NPB, and both were independent of the stage of maturation. *Post hoc* multiple comparisons show that the number of NPBs in female pronuclei was lower than the male in all settings except for cultured embryos at the PN4 stage. * *P* < 0.05, **P < 0.01. The analysis is of 76 and 66 fresh and cultured zygotes, respectively.

Embryos cultured from the PN1 stage also displayed low levels of staining in PN2 zygotes while the levels rose markedly from the PN3 stage (Fig. 1A). The level of staining appeared higher than that in each equivalent stage collected from the reproductive tract. This higher level of staining of cultured embryos showed a similar distribution throughout the nucleoplasm as was observed for freshly collected embryos (Fig. 1B). It was noteworthy that the morphology of the NPBs in the pronuclei appeared to be different in cultured embryos (Fig. 1B), with fewer but larger NPBs in cultured zygotes particularly within the presumptive male pronucleus (Figs 1B and 2).

**Figure 3:**
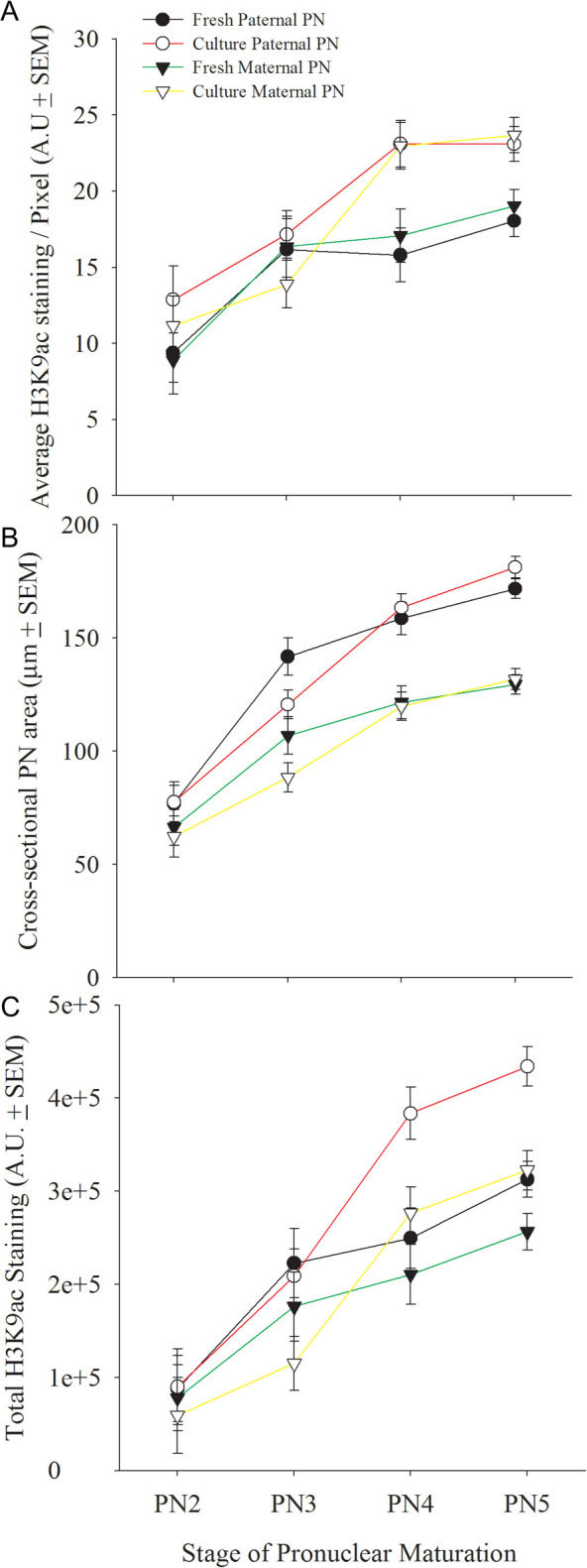
Global H3K9 acetylation levels in the male and female pre-nuclei of zygotic maturation *in vivo* (fresh) or during culture *in vitro*. Zygotes were collected 24 h post hCG (fresh) or 18 h post hCG and cultured for 6 h in KSOM or GE-HTF media (culture). (A) The average staining intensity (A.U., mean ± s.e.m.) across the nucleoplasm at each stage of zygotic maturation. Three-way ANOVA showed that staining intensity increased with stage of development (*P* < 0.001) – this did not differ between maternally and paternally derived pronuclei (***P*** > 0.05), and staining was greater in cultured embryos after PN3 stage (*P* < 0.001). (B) The maximum cross-sectional area of each pronuclei also varied with the stage of maturation. ANOVA showed a significant increase with stage (*P* < 0.001) that differed between the paternal and maternal pronuclei (*P* < 0.004) and was retarded after culture (*P* < 0.006). (C) Total staining across each pronucleus. ANOVA showed a significant increase with stage (*P* < 0.001), and a marked difference between the two pronuclei (*P* < 0.001) which was dependent upon stage (*P* < 0.001). There was no overall effect of culture on total staining (*P* < 0.05). The results are from 4 independent replicates with a total of *n* = 95 fresh zygotes and *n* = 91 cultured zygotes.

Image analysis showed that staining intensity increased at each successive stage of zygotic maturation in both pronuclei in fresh zygotes. Cultured zygotes also had a stage-dependent increased intensity of staining in both pronuclei. The increase in cultured zygotes, however, was greater than that observed in fresh zygotes in the later stages of maturation (PN4–5) (Fig. 3A). Each pronucleus increased in size with progressive development and this increase was greater in the presumptive male pronucleus (Fig. 3B). Culture of zygotes retarded this increase in size but by the PN5 stage there was no effect of culture on pronuclear size (Fig. 3B). The greater size of the presumptive male and female pronuclei resulted in the male pronuclei having higher total staining levels than female pronuclei, and this effect was greater in cultured zygotes (Fig. 3C).

**Figure 4:**
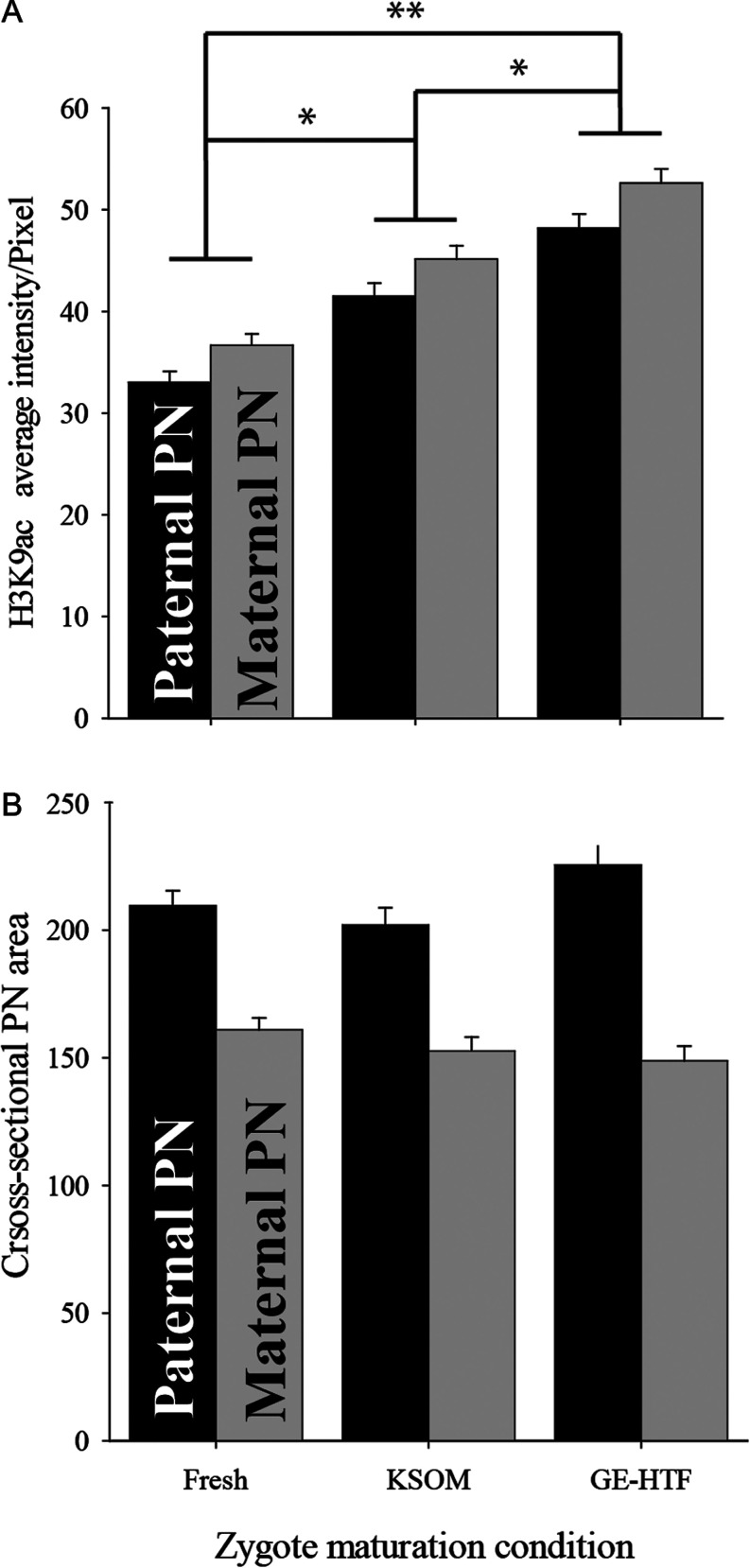
The effect of media formulation on global H3K9ac staining. Zygotes were collected 24 h post hCG (fresh) or 18 h post hCG and cultured for 6 h in KSOM or GE-HTF media. (A) The average H3K9ac staining per pixel (A.U., mean ± s.e.m.) in PN5 zygotes. (B) The equatorial cross-sectional area (µM ± s.e.m.). ANOVA showed that global acetylation levels in male pronucleus was significantly higher in KSOM compared with fresh but significantly less than those in GE-HTF. *P < 0.01, **P < 0.001. Analysis inclusive of 3 independent replicates, Fresh = 18, KSOM = 13, GE-HTF = 11.

GE-HTFM is a minimal essential media for the early embryo. It allows development throughout the preimplantation stage of development in most strains, including those that show a 2-cell block in simpler media (O’Neill 1997). Minimal essential media are important tools for dissecting the essential requirements for growth of given cell types, but in recent years a new generation of media has been developed to optimize long-term development rates following embryo culture. KSOM media supplemented with amino acids (AA) is an example of such media (Biggers & Racowsky 2002). To assess whether media formation contributed to the marked changes in H3K9ac caused by the culture of zygotes, we compared acetylation in GE-HTF compared with KSOM plus AA with zygotes collected directly from the reproductive tract. Culture in KSOM plus AA was also associated with an increased staining intensity, although the extent of this increase was not as great as seen in zygotes cultured in GE-HTFM (Fig. 4A). Neither media type had an effect on pronuclear size (Fig. 4B).

**Figure 5:**
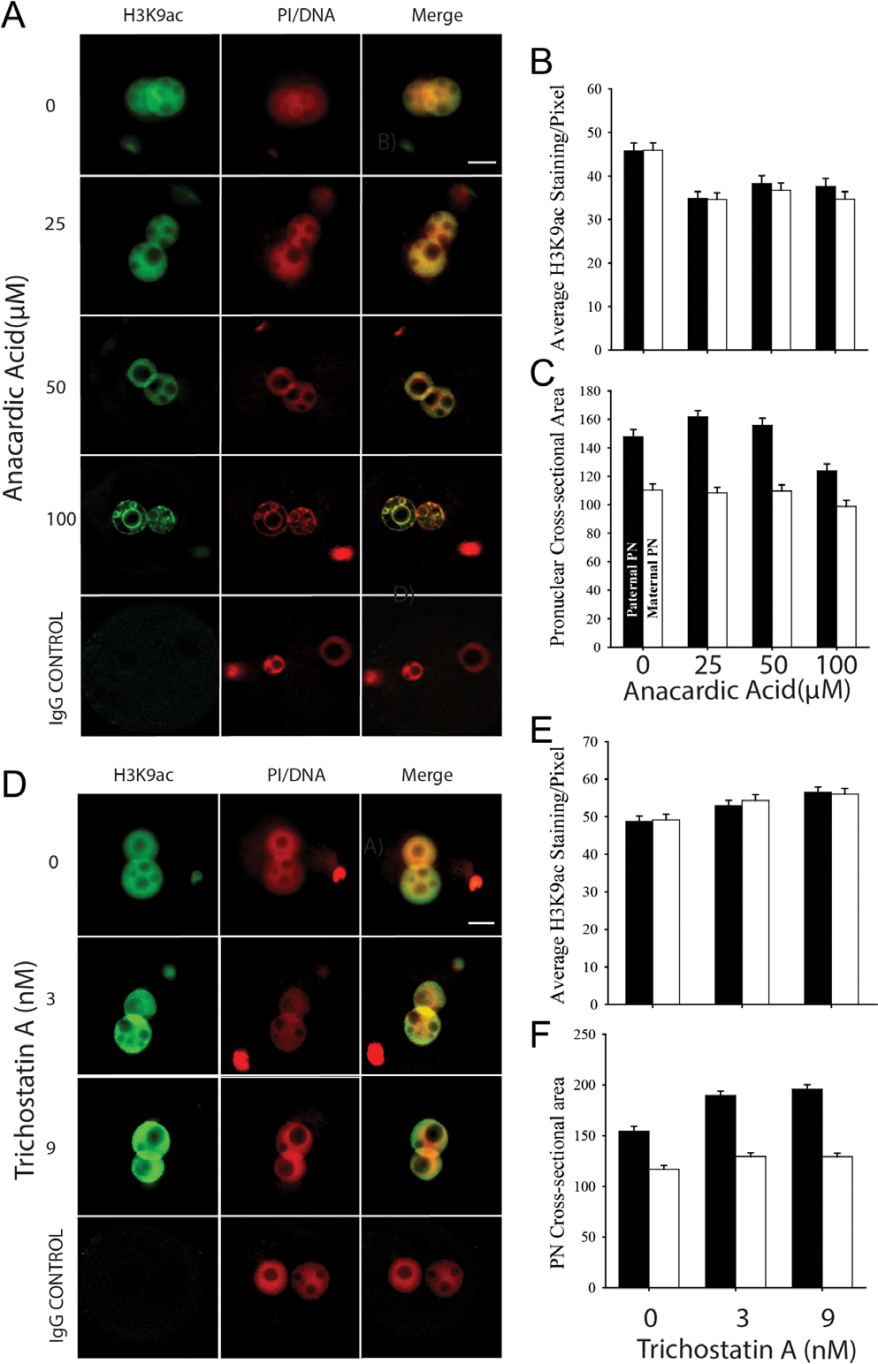
The effect of the HAT inhibitor, anacardic acid, and the HDAC inhibitor, Trichostatin A, on H3K9ac staining and pronucleus size. (A) Zygotes were immunostained for anti-H3K9ac (green) and counterstained for DNA (propidium iodide, PI, red). Whole-section immunofluorescence images were captured and each channel was merged. IgG served as a control. Images are representative of 3 independent replicates with at least 25 zygotes per treatment. Scale bar represents 10 µm. (B) Average H3K9ac staining per pixel (A.U. mean ± s.e.m.) treated with a dose range of anacardic acid. (C) The equatorial cross-sectional area (µm^2^, mean ± s.e.m.) of each pronuclei. ANOVA showed that there was a significant effect of anacardic acid (*P* < 0.01) on staining intensity. This was independent of dose or the parent-of-origin of the pronuclei (*P* > 0.05). There was a significant quadratic effect on paternally derived pronuclei size. The results are from three independent replicates with 50 zygotes per treatment. (D) Whole-section immunofluorescence images of H3K9ac staining after treatment with a dose range of Trichostatin A. (E) The average H3K9ac staining per pixel (A.U., mean ± s.e.m.). (F) Equatorial cross sectional area (µM ± s.e.m.). ANOVA showed there was a significant dose effect on staining level (*P* < 0.001) that was independent of the parent-of-origin of the pronuclei. Trichostatin A caused a significant dose-dependent increase in the size of the pronuclei (*P* < 0.001), and this increase was greater in the paternally derived pronuclei (*P* < 0.01). The results are from three independent replicates with 58 embryos per treatment.

The net level of histone acetylation within the genome results from the equilibrium between the activities of histone acetylases (HATs) and histone deacetylases (HDACs). First we pharmacologically assessed the activity of HATs by the use of a broad-spectrum HAT inhibitor, anacardic acid (Table 1), on H3K9ac in cultured zygotes. Anacardic acid caused a significant reduction in acetylation staining intensity (Fig. 5A and B). It had a complex effect on pronucleus size, causing an increase in the size of the male pronucleus at low concentrations and a decrease in size at the maximum concentration. There was little effect on the size of the female pronuclei except at the maximum concentration (Fig. 5C). The pattern of staining within the nucleoplasm showed a marked change at 100 µM anacardic acid, with an increase in intense regionalized staining and large areas with little staining (Fig. 5A). Inhibition of HDACs using the broad-spectrum inhibitor TSA caused a dose-dependent increase in the intensity of staining in both the male and female pronuclei (Fig. 5D and F) and also a significant increase in pronuclei size, particularly for the male pronucleus (Fig. 5F).

**Table 1:** HAT inhibitors and their targets.

**HAT targeted**	**CBP/p300**	**PCAF**	**GCN5**	**Tip60**
Anacardic acid	Yes	Yes	Yes	Yes
NU 9056				Yes
Butyrolactone			Yes	
Garcinol	Yes	Yes		

**Figure 6:**
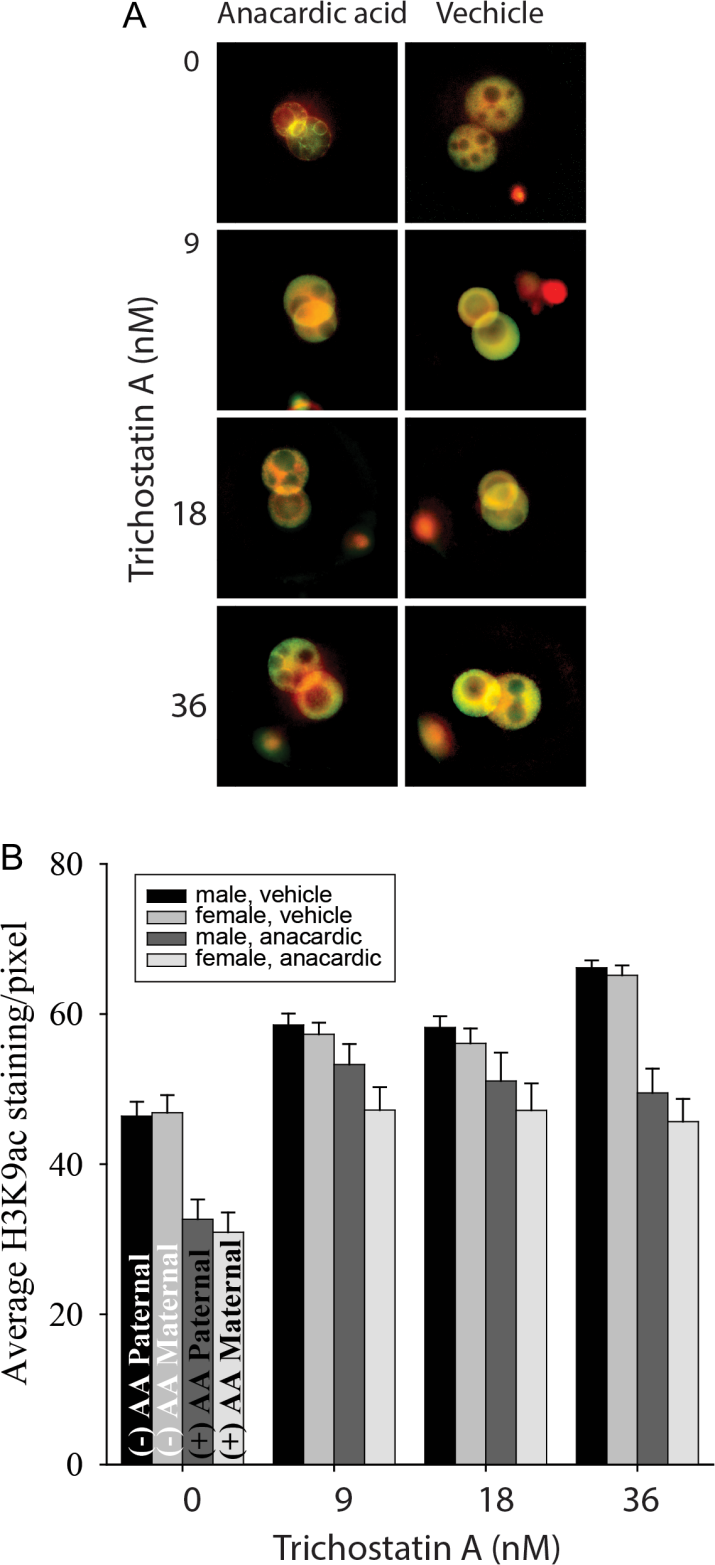
The interaction between HAT and HDAC inhibitors on global levels of H3K9 acetylation in zygotes. (A) Merged images of immunostaining anti-H3K9ac polyclonal antibody (green), counterstained for DNA with PI (red). IgG served as a control. Zygotes were collected from the oviduct at 16.5 h post eCG injection and cultured for 6 h with (100 µM) or without (vehicle) anacardic acid with a graded doses of Trichostatin A. (B) Average staining intensity (A.U., mean ± s.e.m.). The results are of at least 17 zygotes in vehicle and 25 zygotes in anacardic acid at each dose of Trichostatin A. Three-way ANOVA showed that anacardic acid caused a reduction in H3K9ac (*P* < 0.001), while Trichostatin A caused an increase in staining and reversed the effects of anacardic acid (*P* < 0.01). Trichostatin A had a greater effect on the paternally inherited pronuclei (*P* < 0.05).

We next assessed the interactions between HAT and HDAC activity by a factorially designed experiment using both anacardic acid and TSA. This analysis confirmed that anacardic acid alone reduced the staining intensity and TSA alone increased H3K9ac staining intensity. The exposure to both drugs simultaneously, however, showed that TSA could reverse the effects of anacardic acid on the levels of staining and this occurred in both the male and female pronuclei (Fig. 6A and B).

**Figure 7:**
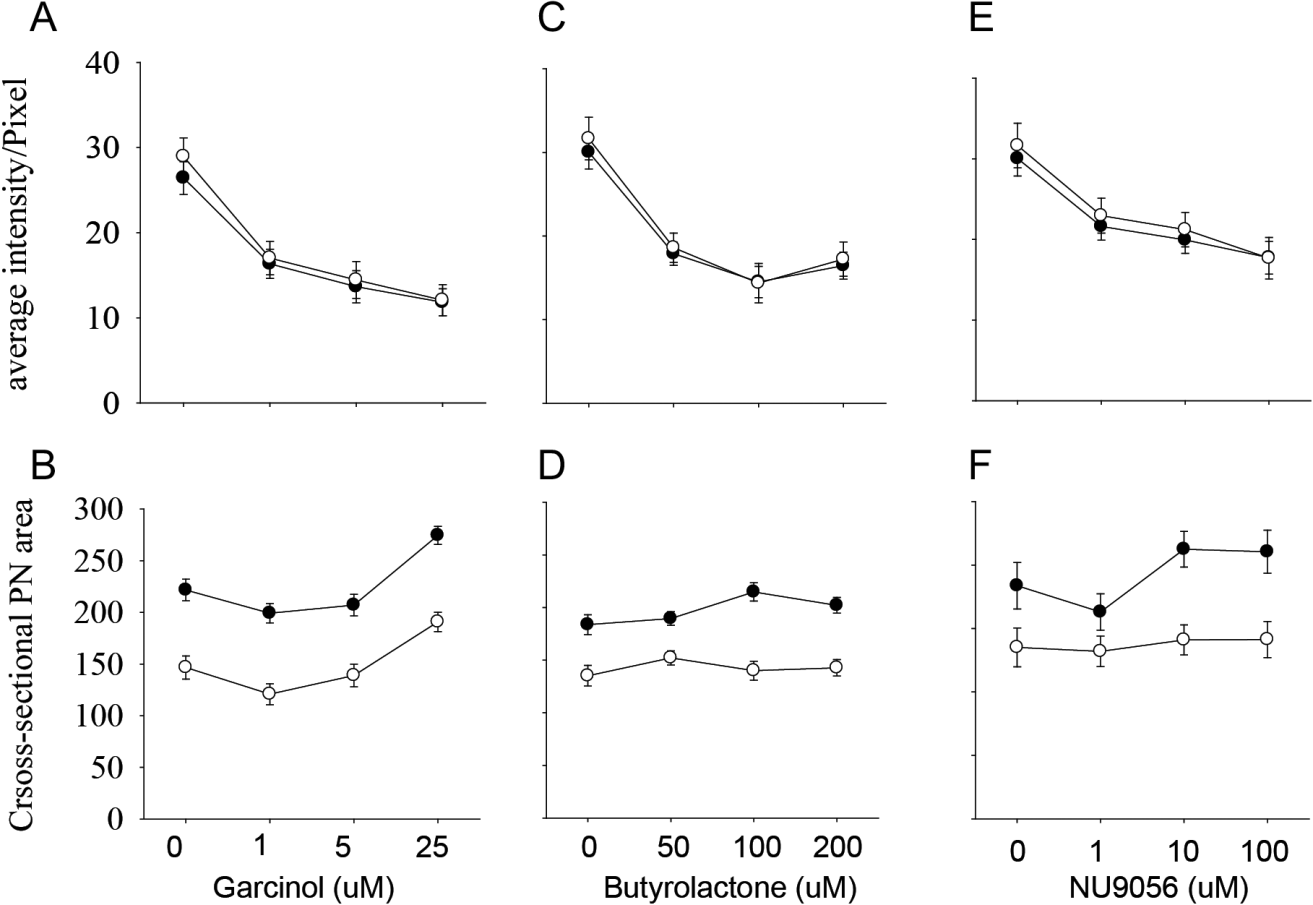
The effect of other HAT inhibitors on H3K9ac acetylation and pronucleus size. The average staining intensity (A.U., mean ± s.e.m.) and pronuclear cross-sectional area (µm^2^, mean ± s.e.m.) in zygotes exposed to increasing concentrations of three structurally and functionally distinct HAT inhibitors (A and B, Garcinol, *n* = 45 zygotes; C and D, Butyrolactone, *n* = 55; and E and F, NU9056, *n* = 42) were measured. Two-way ANOVA showed that Garcinol caused a dose-dependent reduction in acetylation in both and nuclei (*P* < 0.001). It caused a quadratic change in the size of both pronuclei (*P* < 0.01), with a marked increase in size at the highest concentration. Butyrolactone caused a dose-dependent significant reduction in staining in both pronuclei (*P* < 0.001) but had no significant effect on size (*P* < 0.05). NU9056 caused a similar reduction in staining levels (*P* < 0.001) and caused an increase in pronucleus size, but only in the male pronucleus (*P* < 0.01).

To further assess the actions of HATs we screened the effects of a number of other inhibitors with a narrower range of specificity. Despite each of these inhibitors (Garcinol, Butyrolactone and NU9056; Table 1) acting on differing classes of HATs, each caused a significant dose-dependent reduction in the intensity of staining in both the male and female pronuclei (Fig. 7A, B and C). The effects of these HAT inhibitors on pronucleus size was complex, with the general trend being for increased pronuclear size, particularly in the male pronucleus, except for butyrolactone which had no significant effect on size (Fig. 7A, B and C). The results indicate that the global levels H3K9 acetylation observed were the result of the actions of a range of HAT classes.

**Figure 8:**
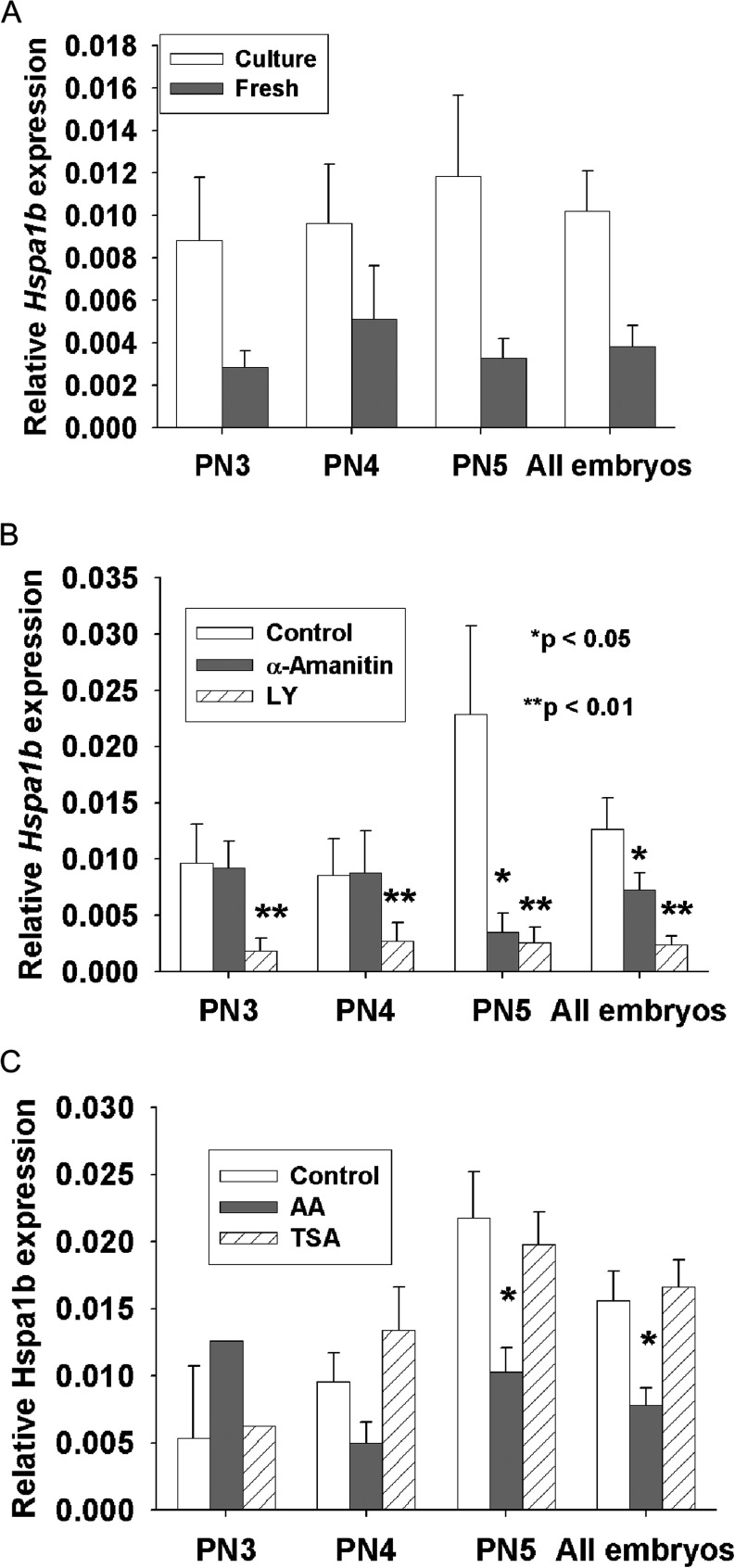
The effect of culture *in vitro* on the onset of expression of *Hspa1b* in the zygote. Zygotes were collected 18 h post hCG and cultured in GE-HTF for six hours, or collected directly from the reproductive tract at 24 h post hCG. The PN stage of maturation of individual zygotes was assessed by Hoechst staining. During culture some zygotes were exposed to inhibitors as shown. (A) The transcript levels of *Hspa1b* are shown relative to the housekeeping gene, *Actb*. The results are the mean ± s.e.m. of 69 and 74 fresh and cultured zygotes, respectively. ANOVA showed that there was no effect of maturation stage on development but cultured embryos had significantly higher transcript levels than fresh controls across all stages (*P* < 0.007). (B) During culture *in vitro* embryos were exposed to the RNA polymerase inhibitor α-amanitin or the PI-3-kinase inhibitor LY294002 (LY). Both caused a significant inhibition of the increased transcript numbers and cultured zygotes; for LY this was evident at all stages but for α-amanitin it was most effective in PN5 zygotes. The results are the mean ± s.e.m. of 42, 41 and 32 zygotes for control, α-amanitin and LY treated zygotes, respectively. (C) Inhibition of HAT (anacardic acid, AA) or HDAC (Trichostatin A, TSA). Anacardic acid caused significant inhibition of HSP 70.1 expression at the PN5 stage (*P* < 0.05), whereas Trichostatin A had no significant effect on transcription (*P* > 0.05). The results are the mean ± s.e.m. of 47, 38 and 53 zygotes in control, and anacardic acid and TSA, respectively.

We next investigated the relationship between these changes in histone acetylation on gene expression from the embryonic genome, using a canonical marker of activation of EGA, *Hspa1b*. The culture of zygotes resulted in significantly higher levels of *Hspa1b* transcripts being detected from the PN3 stage (Fig. 8A). Inhibition of RNA polymerase activity by α-amanitin caused a significant reduction in *Hspa1b* transcripts levels (Fig. 8B). The activity of the phosphatidylinositol-3-kinase signalling pathway is implicated in the activation of transcription and we show that the inhibition of this pathway by LY294002 caused a marked reduction in *Hspa1b* transcript levels (Fig. 8B). Inhibition of HATs by anacardic acid caused a reduction in transcription by the PN5 stage, while treatment with TSA was without effect (Fig. 8C). Increased transcription in cultured zygotes is shown to be dependent upon PI-3-kinase signalling pathways, RNA polymerase activities inhibited by α-amanitin, and the actions of HAT classes that were inhibited by anacardic acid.

## Discussion

Histone acetylation has the potential to markedly change the structure of chromatin and its capacity for gene expression. This study confirms (Wang *et al*. 2007, Bošković *et al*. 2012) that the DNA inherited from the gametes is relatively devoid of H3K9 acetylation but accumulates acetylation as zygotic maturation progresses. This increased acetylation becomes most evident from the PN3 stage of maturation, and this occurs in both pronuclei. The culture of zygotes in simple defined media caused a marked increase in the total global level of acetylation and this affected the male pronucleus more than the female. This created a marked asymmetry in staining between the two pronuclei that was not readily detected in zygotes collected directly from the reproductive tract. The increased level of K3K9 acetylation of each pronucleus caused by culture was reduced when culture was performed in an optimized media. This marked change in acetylation that was readily quantified and clearly affected by the culture conditions points to this change as being a useful potential biomarker for the effects of culture on the normal function of the embryo.

The increased acetylation due to culture caused increased transcription of *Hspa1b*, an accepted marker of the activation of EGA (Christians *et al*. 1995). The increased acetylation and transcription of the embryonic genome were dependent upon the actions of multiple classes of histone acetylases and histone deacetylases. RNA pol II is abundant within the 1-cell embryo (Worrad *et al*. 1995) and it has been proposed that the initiation of EGA may not be due to the activation of transcriptional machinery, *per se*, but rather is the product of changes in chromatin conformation that facilitates accessibility by the transcriptional machinery to gene promoter sites (Worrad *et al*. 1995). Such a mechanism has been reported in pluripotent embryonic stem cells (ESCs), where H3K9 and K14 acetylation establish an open chromatin structure (Karmodiya *et al*. 2012) and enable RNA pol II docking at actively transcribed gene loci (Karmodiya *et al*. 2012). Thus, an increase in H3K9ac concomitant with DNA replication (PN3–4) (Santos *et al*. 2013) during the zygotic cell cycle may foster gene transcription by providing easily assessable binding sites for RNA pol II at gene loci (Fillingham & Greenblatt 2008). Our observation of the reversible effects of H3K9ac on transcription of *Hspa1b* is consistent with this hypothesis. Of course H3K9 is only one potential target for histone acetylation within the genome and the effect of culture on the range of other histone modifications is an important future priority.

Asymmetric epigenetic modifications between the parental genomes during zygotic maturation have been reported and include DNA methylation (Oswald *et al*. 2000), H3K9 methylation, H3K27 methylation (Santos *et al*. 2005) and H4K5 acetylation (Adenot *et al*. 1997). Here we show a symmetrical pattern of H3K9ac staining between both pronuclei in zygotes collected directly from the reproductive tract, yet culture caused a shift to greater staining in the male pronuclei and a resulting asymmetry. It is of interest to note that culture also accounts for reports of asymmetric DNA methylation between the male and female pronuclei (Li & O’Neill 2012, 2013).

A characteristic feature of the architecture of pronuclei during zygotic maturation is their progressive expansion in size. This expansion is considered to be due in part to decompaction of chromatin structure, and histone acetylation is well known to foster a more open chromatin conformation (Grunstein 1997, DesJarlais & Tummino 2016). The parallel between the increase in H3K9ac staining and pronuclear size may therefore be suggestive of a causative relationship. Yet the studies using inhibitors of HATs and HDAC indicate a complex association between acetylation and pronuclear size. The increased acetylation caused by inhibition of HDACs was associated with increased pronuclear size. It is important to be mindful however that this treatment will inhibit most histone acetylases so this result does not of itself show a role for H3K9 acetylation in this expansion. Furthermore, the inhibition of HATs that are more targeted to H3K9 had less consistent effects. For example, butyrolactone caused a marked reduction in acetylation without causing a decrease in pronuclear size.

Mechanistically, the relative hyperacetylation of H3K9 induced by culture suggests that there may be an alteration in the relative contribution of HAT or HDAC activity, resulting in a higher net level of acetylation in the *in vivo* zygotes compared to those that develop in the reproductive tract. The large number of potential histone acetylases and histone deacetylases that exist in cells prevented strict characterisation of the particular enzymes involved but the pharmacological screen implicates the activity of a range of HATs. TSA inhibits a wide range of HDACs; in line with previous reports (Yoshida *et al*. 1990) this study used nanomolar concentrations of TSA which are considered to exert selective effects on HDACs. AA is a reported to have an IC_50_ of >200 µM for PCAF/GCN5 and CBP/p300 and 64 µM for Myst protein Tip60 (Ghizzoni *et al*. 2012). Garcinol has an IC_50_ of 3.2–21.4 µM (Hong *et al*. 2007). Butyrolactone has an IC_50_ of 100 μM (Biel *et al*. 2004). NU 9056 has an IC_50_ of <2 μM (Coffey *et al*. 2012). Thus, these drugs reduced HeK9ac in the zygote at concentrations where they were expected to have selective actions against HATs. The large number of HATs and HDAC that exist, and the very small amount of biological material available for analysis in the zygote precluded detailed biochemical characterisation of the particular enzymes involved in this study. However, this pharmacological analysis provides clear evidence of the action of a range of enzymes and can be used to guide future investigation into the effectors.

It is now generally recognized that culture of zygotes *in vitro* can increase epigenetic instability in progeny and this is particularly evident for metastable epialleles (Mahsoudi *et al*. 2007, Morgan *et al*. 2008, Fernandez-Gonzalez *et al*. 2010). This instability of metastable epialleles stems from their incomplete reprogramming during the preimplantation stage of development. Detailed analysis of the *Axin1^Fu^* epiallele showed that increasing histone acetylation at the zygote stage by transient treatment with an HDAC inhibitor phenocopied the epigenetic instability at this allele caused by zygote culture (Fernandez-Gonzalez *et al*. 2010). Our finding that culture increased H3K9ac levels in the zygote, the reported increased epigenetic instability of cultured zygotes (Mahsoudi *et al*. 2007, Morgan *et al*. 2008, Fernandez-Gonzalez *et al*. 2010), and evidence that H3K9ac may be a reprogramming mark that determines the epigenetic state of a metastable epiallele (Fernandez-Gonzalez *et al*. 2010) place this histone modification as a potential important regulator of epigenetic reprogramming during early embryo development.

The study points to a role of H3K9 acetylation in the maturation processes of the zygotic pronuclei that readies them for the initiation of embryonic genome activation. It shows that culture of embryos *in vitro* can cause marked changes in the dynamics of this acetylation. This in turn influences the normal level of gene expression from the zygotic genome and may influence epigenetic stability postnatally. These changes may serve as a biomarker for assessing the optimal conditions for embryo development and also point to dangers in using cultured embryos to draw conclusions about the normal molecular and epigenetic processes of embryo development.

## Declaration of interest

The authors declare that there is no conflict of interest that could be perceived as prejudicing the impartiality of the research reported.

## Funding

This work was supported by grants from the Australian National Health and Medical Research Council.
